# The value of linear and non-linear quantitative EEG analysis in paediatric epilepsy surgery: a machine learning approach

**DOI:** 10.1038/s41598-024-60622-5

**Published:** 2024-05-13

**Authors:** Mattia Mercier, Chiara Pepi, Giusy Carfi-Pavia, Alessandro De Benedictis, Maria Camilla Rossi Espagnet, Greta Pirani, Federico Vigevano, Carlo Efisio Marras, Nicola Specchio, Luca De Palma

**Affiliations:** 1 Neurology, Epilepsy and Movement Disorders Unit, Bambino Gesù Children’s Hospital, IRCCS, Full Member of European Reference Network EpiCARE, Piazza S. Onofrio 4, 00165 Rome, Italy; 2 Department of Physiology, Behavioural Neuroscience PhD Program, Sapienza University, Rome, Italy; 3 Neurosurgery Unit, Bambino Gesù Children’s Hospital, IRCCS, Full Member of European Reference Network EpiCARE, 00165 Rome, Italy; 4 Neuroradiology Unit, Imaging Department, Bambino Gesù Children’s Hospital, 00165 Rome, Italy; 5 Department of Mechanical and Aerospace Engineering – DIMA, Sapienza University of Rome, Rome, Italy

**Keywords:** Resective surgery, Epilepsy, Predictive factors, Diagnostic evaluation, Scalp EEG, Brain machine learning, Non-linear EEG biomarkers, Entropy, Lyapunov exponents, Hjorth parameters, Hurst index, Artificial neural network, Feature selection, Seizure outcome, Computational neuroscience, Diseases of the nervous system

## Abstract

Epilepsy surgery is effective for patients with medication-resistant seizures, however 20–40% of them are not seizure free after surgery. Aim of this study is to evaluate the role of linear and non-linear EEG features to predict post-surgical outcome. We included 123 paediatric patients who underwent epilepsy surgery at Bambino Gesù Children Hospital (January 2009–April 2020). All patients had long term video-EEG monitoring. We analysed 1-min scalp interictal EEG (wakefulness and sleep) and extracted 13 linear and non-linear EEG features (power spectral density (PSD), Hjorth, approximate entropy, permutation entropy, Lyapunov and Hurst value). We used a logistic regression (LR) as feature selection process. To quantify the correlation between EEG features and surgical outcome we used an artificial neural network (ANN) model with 18 architectures. LR revealed a significant correlation between PSD of alpha band (sleep), Mobility index (sleep) and the Hurst value (sleep and awake) with outcome. The fifty-four ANN models gave a range of accuracy (46–65%) in predicting outcome. Within the fifty-four ANN models, we found a higher accuracy (64.8% ± 7.6%) in seizure outcome prediction, using features selected by LR. The combination of PSD of alpha band, mobility and the Hurst value positively correlate with good surgical outcome.

## Introduction

Resective surgery is highly effective in selected subgroups of patients with medication-resistant seizures^1,2^, and between 40 and 80% of patients achieve complete post-operative seizure freedom (SF)^1–3^. Aetiology is the main predictor of post-surgical outcome^3^. A better understanding of the possible predictive value of pre-surgical investigations may improve surgical outcome^4,5^. A comprehensive diagnostic work-up with information coming from multiple modalities as electroencephalography (EEG), magnetic resonance imaging (MRI), neuroimaging post-processing and functional neuroimaging may estimate the likelihood of post-surgical seizure freedom, even if the weight of each investigation in determining the outcome is not completely understood^6,7^. Patients with clearly localized epileptogenic zone and concordance between multiple diagnostic modalities have a higher rate of seizure freedom^8^. Scalp EEG is a standard procedure used to localize the epileptogenic zone^2^, suffering from discordant results^7,9^. Quantitative analysis of pre-surgical scalp EEG may help in identifying patients with higher likelihood of seizure freedom, even if such a methodologic approach is still underused^10^ . The use of quantitative scalp EEG analysis is challenging due to the common presence of background noise, artefacts and the signal’s non-stationarity nature^11^.

Traditional EEG analysis methods primarily focus on linear features such as amplitude and frequency, which have limited ability to capture the complex dynamics of the signal^12^. Non-linear EEG features, like Lyapunov Exponent and Entropy, provide a more comprehensive analysis of the EEG signal by capturing the underlying dynamics of the signal^13^. Interictal linear and non-linear EEG analysis may disclose pro-epileptogenic regions^14–16^. These analysis are mainly used to predict epileptic seizures and not surgical outcome^17^. Entropy^18,19^ and Lyapunov exponents^20^, can be used to quantify the level of epileptogenicity examining the brain’s predictability and chaos levels.

Non-linear EEG features derived from EEG rhythms and wavelet decomposition, achieve a high prediction accuracy (85–93%) in detecting focal versus non-focal epilepsies^21^. Other linear and non-linear biomarkers, such as Hjorth parameters^22^ and Hurst index^23,24^, may provide an estimate of the dynamic interaction in different scalp EEG signals, and may predict seizure lateralization^22^. Power spectral density, hjorth, approximate entropy, permutation entropy, Lyapunov and Hurst exponent in scalp EEG are limited by artefacts, low spatial resolution and low sampling^25^.

Brain machine learning (BML) is a powerful tool in biomedical applications often underused in EEG analysis^26^. BML methods are used for automated epilepsy diagnosis prediction^27^, seizure detection (accuracy 57–100%)^28^ and localization (accuracy 93–96%)^29^.

No studies have demonstrated how BML algorithms can be employed to evaluate the role of a specific combination of linear and non-linear interictal EEG features to predict surgical outcome.

Aim of this study is uncover hidden EEG features that may contribute to the variability of epilepsy surgery outcomes, computing and integrating linear and non-linear features in awake and sleep scalp and interictal EEG signals and applying a BML approach.

## Results

**Figure 1:**
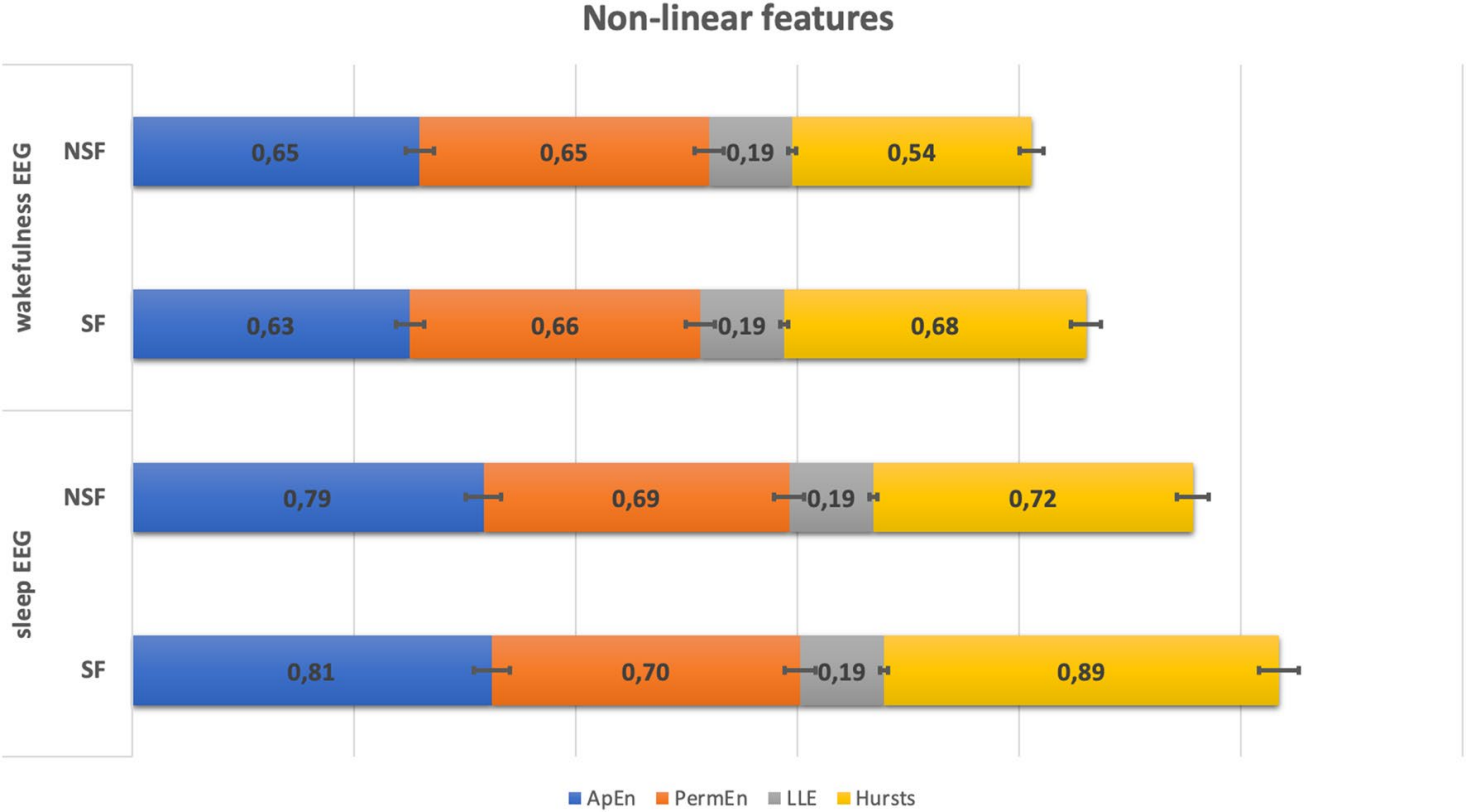
Mean values of Hurst, ApEn, PermEn and LLE non-linear EEG features extracted for both sleep and wakefulness condition: comparison between SF and NSF surgery outcome values. ApEn, approximate entropy; PermEn, permutation entropy; LLE, Lyapunove Exponent.

We screened 246 interictal scalp recordings (2 EEGs for each patient, one recording during wakefulness and one during sleep) from 123 patients. All EEGs were free from artifact and epileptiform abnormalities. We collected 1-min of each EEG to extract linear and nonlinear features. The descriptive analysis of the linear and nonlinear EEG features is summarized in Supplementary Table 1. The descriptive comparison of non-linear EEG features are represented in Fig. 1.

The results of logistic regression (LR) test showed that Hurst value during sleep EEG is positively correlated with seizure freedom (OR = 2.681, 1.084 < CI < 6.629, *p* = 0.033), while Hurst value during wakefulness EEG was negatively correlated with seizure freedom (OR = 0.281, 0.082 < CI < 0.967, *p* = 0.044).

Power spectral density (PSD) in alpha band during sleep (OR = 1.400, 1.001 < CI < 1.007, *p* = 0.019) and mobility index during sleep (OR = 2.783, 1.140 < CI < 6.797, *p* = 0.025) were positively correlated with seizure freedom. These statistically significant EEG features were used in SET 3. No statistical correlation was found between Hjorth, approximate entropy, permutation entropy, and Lyapunov indexes and surgical outcome (*p* > 0.05).

**Figure 2:**
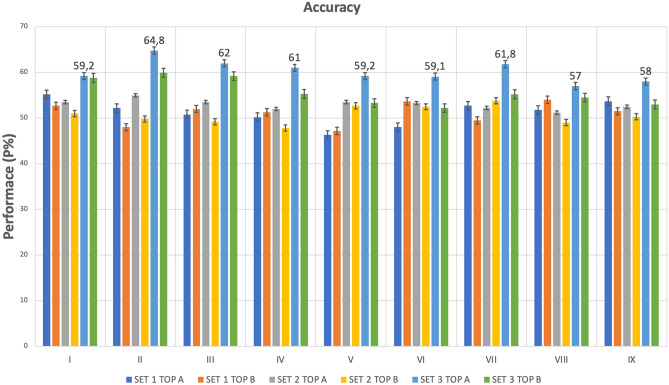
Comparison of prediction accuracy (P%) generated by different ANN architectures (I–IX), SET of features (1–3) and ANN topology (A–B). The best accuracy results are related to Set 3-II ANN architecture, Topology A and SET 3 (light-blue bar) were the best combination of SET and topology in terms of accuracy. SET 3 with topology A led to a higher predictive value for all ANNs (P% = 57–64.8%) compared to SET 1 and SET 2.

The results of accuracy of each artificial neural network (ANN) using SET 1 (all features), SET 2 (averaged features) and SET 3 (features selected by LR) as training sets are reported in Table 1. The comparison of prediction accuracy (P%) generated by different ANN architectures (I-IX) and SET (1–3) of features is shown in Fig. 2.

**Table 1:** Results of accuracy, sensibility and specificity of the nine artificial neural networks architectures, topology and SET of features used as input of models.

Sets	Architecture	Topology A				Topology B			
		Nmax	Accuracy P (%)	Sensibility (%)	Specificity (%)	Nmax	Mean accuracy P (%)	Sensibility (%)	Specificity (%)
Set1	I	45	55.2 ± 7.7	87.3 ± 7.3	23.0 ± 14.2	40	52.7 ± 9.3	79.3 ± 13.5	26.0 ± 15.1
	II	45 + 45	52.2 ± 6.6	89.3 ± 10.5	15.0 ± 1.1	40 + 20	48.0 ± 6.9	80.0 ± 14.7	16. 0 ± 9.7
	III	45 + 45 + 45	50.8 ± 4.5	90.7 ± 10.5	11.0 ± 9.9	40 + 20 + 10	52.0 ± 5.5	88.0 ± 13.6	16.0 ± 19.6
	IV	40	50.2 ± 8.7	85.3 ± 5.3	15.0 ± 16.5	30	51.3 ± 7.7	90.7 ± 15.1	12.0 ± 19.9
	V	40 + 40	46.3 ± 6.5	82.7 ± 11.8	10.0 ± 6.7	30 + 15	47.2 ± 7.7	81.3 ± 15.7	13.0 ± 6.7
	VI	40 + 40 + 40	48.0 ± 3.3	84.0 ± 9	12.0 ± 13.2	30 + 15 + 8	53.7 ± 7.2	81.3 ± 10.8	26.0 ± 17.8
	VII	35	52.7 ± 7.8	77.3 ± 14.1	28.0 ± 11.4	25	49.5 ± 4.4	80.0 ± 16.3	19.0 ± 11
	VIII	35 + 35	51.8 ± 10.4	80.7 ± 13.5	23.0 ± 18.9	25 + 12	54.0 ± 7.3	82.0 ± 17.5	26.0 ± 20.1
	IX	35 + 35 + 35	53.7 ± 3.3	83.3 ± 16.4	24.0 ± 20.7	25 + 12 + 6	51.5 ± 5.2	86.0 ± 10.2	17.0 ± 8.2
Set2	I	36	53.5 ± 8.7	84.0 ± 11.4	23.0 ± 18.3	36	51.0 ± 9.7	78.0 ± 14.4	24.0 ± 11.7
	II	36 + 36	55.0 ± 13.3	82.0 ± 13.7	28.0 ± 22	36 + 18	49.8 ± 5.7	78.7 ± 15.7	21.0 ± 16
	III	36 + 36 + 36	53.5 ± 6.2	80.0 ± 16.6	27.0 ± 17	36 + 18 + 9	49.2 ± 7.4	87.3 ± 15.5	11.0 ± 7.4
	IV	30	52.0 ± 5.7	82.0 ± 13.7	22.0 ± 13.2	30	47.8 ± 6.6	70.7 ± 21.4	25.0 ± 23
	V	30 + 30	53.5 ± 8.1	80.0 ± 18.1	27.0 ± 15.7	30 + 15	52.7 ± 7.7	87.3 ± 11.5	18.0 ± 14.8
	VI	30 + 30 + 30	53.3 ± 11.9	76.7 ± 11.9	30.0 ± 20	30 + 15 + 8	52.5 ± 11	80.0 ± 18.3	25.0 ± 17.2
	VII	20	52.2 ± 10	83.3 ± 15.8	21.0 ± 12	20	53.8 ± 6.2	80.7 ± 18.4	27.0 ± 21.1
	VIII	20 + 20	51.2 ± 10.2	85.3 ± 14	17.0 ± 15.7	20 + 10	49.0 ± 6	80.0 ± 12.6	18.0 ± 12.3
	IX	20 + 20 + 20	52.5 ± 9.7	88.0 ± 12.1	17.0 ± 20	20 + 10 + 5	50.3 ± 12.2	70.7 ± 10.5	30.0 ± 19.4
Set3	I	9	59.2 ± 7.9	79.3 ± 15.2	25.0 ± 15.8	9	58.8 ± 7.1	76.7 ± 14.8	31.0 ± 18.5
	II	9 + 9	64.8 ± 7.6	76.7 ± 14.1	43.0 ± 14.2	9 + 5	59.3 ± 7	84.7 ± 15.1	20.0 ± 18.9
	III	9 + 9 + 9	62.0 ± 7.9	76.0 ± 11.8	24.0 ± 19.6	9 + 5 + 3	59.2 ± 7.7	61.3 ± 17.7	43.0 ± 26.3
	IV	6	61.0 ± 6.4	80.0 ± 11.8	22.0 ± 18.7	6	55.3 ± 9.8	84.7 ± 13.4	26.0 ± 16.5
	V	6 + 6	59.2 ± 6.8	79.3 ± 15.5	21.0 ± 12.9	6 + 3	53.3 ± 5.6	80.7 ± 27.7	26.0 ± 32
	VI	6 + 6 + 6	59.1 ± 6.2	67.3 ± 11.1	31.0 ± 11	6 + 3 + 2	52.2 ± 6.4	91.3 ± 8.3	13.0 ± 17
	VII	4	61.8 ± 6.8	84.7 ± 7.7	19.0 ± 9.9	4	55.2 ± 6.2	87.3 ± 7.3	23.0 ± 11.6
	VIII	4 + 4	57.0 ± 9.9	82.0 ± 16.6	32.0 ± 18.1	4 + 2	54.5 ± 10.4	82.0 ± 16	27.0 ± 27.5
	IX	4 + 4 + 4	58.0 ± 10.1	82.0 ± 9.5	24.0 ± 18.54	4 + 2 + 1	53.0 ± 7.7	78.0 ± 17.2	28.0 ± 21

The best classification performance is related to Set 3-II ANN architecture, topology A (64.8%). The best model had a 76.7% sensitivity and 43% of specificity, which was higher than other architectures tested. Nmax, maximum number of neurons in each hidden layer used.

Analysing the results of BML (Table 1 and Fig. 2), we found that the II architecture of ANN was the most accurate in predicting seizure outcome, with a specific topology of 2HL and 9 N, and a specific set of EEG features (SET 3). This combination resulted in the highest prediction accuracy (*p* = 64.8% ± 7.6). The optimal combination of EEG features is presented as linear combination of 4 linear and non-linear EEG features, including Hurst during wakefulness and sleep recordings, Mobility during sleep EEG, and PSD in alpha band during sleep EEG.

SET 3, represented by the light-blue bar, emerged as the most effective training set across all tested ANN architectures, as showed in Fig. 2. When combined with topology A (Fig. 1), SET 3 achieved superior predictive accuracy for all ANNs architectures, with accuracy values ranging from 57 to 64.8%. The use of SET 3 outperformed both SET 1 and SET 2. The analysis revealed no notable differences in predictive performance between SET 1 and SET 2. Similarly, no significant distinctions were observed among the various topologies of the ANNs. (*p* > 0.05, CI = 95%).

The best model, featuring II ANN architecture with topology A and SET 3, demonstrated superior prognostic accuracy in identifying SF outcomes, achieving a notable sensitivity of 76.7% and a specificity of 43%, exceeding the performance metrics of all other models tested.

The specificity of best model was the highest compared to all architectures using SET 3.

Comparing the performance of different architectures (from I to IX) related to the best model (II architecture, SET 3 and topology A) we found that the accuracy was significantly higher than all other combinations of topology and SET (*p* value < 0.05, CI = 95%).

## Discussion

We explored the role of EEG dynamic properties in predicting epilepsy surgery outcome. Currently only clinical, neuroimaging and neurophysiological qualitative predictors^1,3,9^ are strongly correlated with post-surgical seizure outcome, moreover only some of these predictors are used to generate predictive models of seizure outcome^9^. To characterize EEG segments, we composed three different sets (SET1, SET2, SET3) of EEG features as descripted in Methods section. We applied two different approaches. To compute SET 1 we performed a single channel extraction: all predicting values are computed at each channel of standard 10–20 montage and epoch as reported in Lemoine et al.^30^. To compute SET 2 and 3 we extracted all values as the average across all channels according to Lin et al.^31,32^. This approach may mitigate the variability in single-channel data.

We used selected features for the SET 3 which were extracted using LR, and we achieved the best accuracy in predicting surgical outcome. Among power spectral density, Hjorth, approximate entropy, permutation entropy, Lyapunov and Hurst exponent averaged EEG features, some may be redundant or may not contain enough discriminative information for the prediction^33^. In addition, the ANN with a high number of EEG features may be influenced by the relatively low number of patients^34^.

Hurst exponent is the only non-linear EEG feature able to discriminate between SF and non-Seizure freedom (NSF) patients. LR analysis demonstrated that increasing the regularity (increasing Hurst value) of the EEG signal during sleep, the chances of seizure freedom increased (OR = 2.681, 1.084 < CI < 6.629 with *p* = 0.033), while increasing the regularity of the EEG signal during wakefulness, the chances of seizure freedom decreased (OR = 0.281, 0.082 < CI < 0.967 with *p* = 0.044).

The LR considers the distribution of values and their relationship with the outcome variable, rather than a simple comparison of mean values. An increase in Hurst values in wakefulness is associated with a lower probability of attaining seizure freedom. On the other hand in sleep a "positive correlation" is an increase in Hurst values associated with a higher probability of achieving seizure freedom. A study by Witton et al.^35^ analysed Hurst exponent of pre-surgical EEG signals and found that Hurst value was able to identify the probable epileptogenic zone in 3 out of 3 patients (100%). The interpretation of Hurst values can be challenging, as they are affected by signal length, noise level, and sampling rate^36^.

Alpha band PSD is a potential biomarker for the automatized detection of epileptic seizures, achieving a 98% of accuracy model^37^, even if alpha band PSD analysis of EEG signals is affected by the total power of the spectrum^38^. In our study Alpha band PSD during sleep stage is positively correlated with seizure outcome (OR = 1.400, 1.001 < CI < 1.007 with *p* = 0.019). No previous studies demonstrated the value of alpha band PSD in predicting epilepsy surgical outcome in a paediatric population, differently changes in the alpha band PSD have been correlated with several neurological and psychiatric diseases in adults^39^.

Mobility had only been studied in seizure prediction and lateralization^40^. In addition, C.S Ouyang et al.^41^ observed a significant increase of mobility in patients who benefit from anti-seizure medications. In the present study, the mobility index calculated during the sleep state was correlated with the post-surgical outcome indicating that higher value significantly improves the probability of seizure freedom (OR = 2.783, 1.140 < CI < 1.6.797 with *p* = 0.025). These results confirm our previous study showing that the mobility index positively correlated with favourable surgical outcome in patients undergoing hemispherectomy (*p* = 73%)^42^.

We then focused on the specific combination of linear and non-linear EEG features to predict surgical outcome.

Lemoine et al. investigated how combining linear and non-linear EEG features could predict seizure recurrence within 1 year after EEG, using four BML algorithms (general linear model, support vector machine, Random Forest and LightGBM). They achieved an accuracy rate between 62 and 67%^30^. Previously, no studies had showed that such a combination of linear and non-linear interictal scalp EEG features could accurately predict surgical outcome in children with epilepsy. In our three SETs of EEG features, SET 3 showed the most promising results revealing the lowest mean square error (MSE = 9.2). This indicates that our choice of features and the size of our dataset were well-suited for creating a stable model^30,42,43^.

In the last few years more and more studies tried to predict the post-surgical outcome using predictive models. The best results were achieved using clinical variables. Grigsby et al. trained an ANN classifier using clinical, neuropsychological and imaging data from 65 patients treated with anterior temporal lobectomy; the accuracy was of 81.8% in predicting Engel I outcome (improving to 95.4% for Engel I or II outcomes)^4^. Arle et al. also applied ANN with several architecture, reporting an accuracy of 96% in predicting Engel I outcomes in unselected 80 surgical patients^44^. Different methods have been utilized (nomograms and simple seizure-freedom scores) to predict seizure freedom in mixed adult and paediatric populations^1,45^ with poor predictive value (AUC of 0.528–0.539 and 0.533–0.539 at 2 and 5 years time points)^46^. Sinclair et al. evaluated, also, the potential of BML techniques applied to standard presurgical brain MRs and PET scans to provide enhanced prognostic value to such neuroimaging tools. Up to 73% of patients with poor surgical outcome were predicted, potentially providing additional information to incorporate into surgical decision-making process^47^.

The choice of specific BML tool and the number of architectures and topology are still not properly defined. We do not have a pre-defined model with fixed number of architectures. Our best model (II—SET3—topology A) showed a significant higher accuracy than all other combinations of topology and SETs (*p* value < 0.05). We observed that, using SET 3, the ANN performed better than using SET 1 (all features) and SET 2 (averaged), as it is shown in Table 1. The choice to use 3 training SETs was arbitrary. Each set of features might involve different levels of processing leveraging the strengths of each of them^48^.

Our findings suggest that a specific architecture and specific selection of EEG features may improve ANN performance, indicating that not all EEG features are effective in predicting epilepsy surgical outcome. Similar results were found in a previous study which demonstrated that some clinical and EEG features are irrelevant for prognosis. In this study three BML models (Naïve Bayes, logistic regression and K-NN) were used: authors extracted from 23 patients with Hippocampal Sclerosis a specific set of features achieving an improvement of accuracy from 68.42 to 89.47%^49^.

Accurate prediction of seizure outcome after epilepsy surgery remains difficult. traditional statistical modelling (LR) and machine learning techniques (multilayer perceptron and XGBoost) performed equally (72% vs 71%, *p* > 0.05) to predict 1-year post-operative seizure outcome on 797 children who undergone resective or disconnective surgery^50^.

We do have some limitations to be acknowledged. We were missing an external validation cohort, and this could lead to underperform if the same model is tested on data coming from a different sample^50^. Despite this limitation, the ANN may provide a powerful tool to optimize patient management^51,52^ improving the inherent characteristics and quality of data. As previously recommended^50^, we also strongly believe that a collaboration to create standardized datasets, selection of appropriate predictor variables for modelling, sharing of models and code, are essential for advancing this research field. It is important to note that in our study we used only scalp EEG signals, which are known to have lower spatial resolution and high level of signal noise^53,54^, if compared with to Stereo-EEG signals. Moreover, the performance of the ANN model may be affected by the dataset size and the monocentric recruitment. Future studies may also consider interpolating other types of data, such as neuroradiological and clinical features, to improve the accuracy of the ANN. Furthermore, our study included only interictal scalp EEG segments free from epileptiform abnormalities, and it may turn that EEG signal with epileptiform abnormalities can discriminate better dynamical EEG properties^55^. Despite these limitations, our results suggest that this ANN model may hold considerable promise as adjuncts to clinical expertise and not as a replacement.

Our study is the first to investigate the relationship between linear and non-linear EEG properties and surgical outcomes in paediatric epilepsy patients. We found that a specific combination of EEG features, such as the Hurst exponent, Mobility Index and PSD, were correlated with post-surgical seizure freedom, achieving an accuracy of 64.8% in predicting surgical outcome. The main contributions of this study are: (1) the first development of an automated and quantitative approach and tool for early prediction of epilepsy based on interictal EEG classification analysis; (2) identification of significant linear and non-linear EEG features for discriminating between SF and NSF patients.

## Methods

We retrospectively reviewed all paediatric patients who underwent surgery for medication-resistant epilepsy from January 2009 to April 2020, at Bambino Gesù Children’s Hospital, Rome, Italy. All methods were carried out in accordance with relevant guidelines and regulations. Ethical approval and inform consent were waiver by ethical committee of the Ospedale Bambino Gesu’. Data were retrospectively analysed in line with personal data protection policies.

We found 169 patients. We included only patients with the following inclusion criteria:

1. Available pre-surgical EEG data during wakefulness and sleep.
2. One-minute artefact free awake and sleep EEG.
3. Post-surgical follow-up of at least 3 years.

One-hundred-twenty-three patients were enrolled in the study. The mean age at surgery is 7.3 ± 12.2 years, 73 out of 123 (59.4%) patients are seizure free and drug free (SF) and never experienced post-surgical seizures; 50 out of 123 (40.6%) are non-seizure free (NSF). Supplementary Table 2 show the results of histopathology examination on brain specimen. All patients underwent routine pre-surgical evaluation, including full history and neurological examination, brain MRI and visual analysis of long-term video-EEG monitoring. All patients underwent neuropsychological assessment during follow-up.

**Figure 3:**
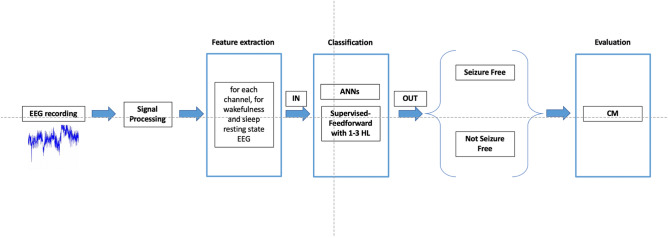
Block-diagram of the proposed surgical outcome prediction method using artificial neural network (ANN). HL, hidden layers; CM, confusion matrix.

The study includes six stages (Fig. 3): EEG recording, signal processing and analysis, features extraction and selection, and classification. EEG recordings were obtained with a VEEG monitoring system (Micromed, Treviso, Italy) at the Neurology, Epilepsy and Movement Disorders Unit of the Bambino Gesù Children’s Hospital in Rome, Italy. The signal processing, analysis and classification were computed with MATLAB software (R2022b). The 10–20 electrode montage was used for scalp recordings.

The reference electrode was set as the average of all contacts. The monopolar recordings were obtained with a sampling frequency of 256 and 512 Hz, powerline notch filtered at 50 Hz, band-pass filtered between 0.5 and 45 Hz (7th order Butterworth filter) and 16-bit resolution and z-score standardized.

The extraction of EEG data was performed primarily by neurophysiology expert (CP, GCP) through visual inspection. Before filtering, the EEG signals were down sampled (256 Hz). Sixty seconds of EEG signal was used both in wakefulness and sleep. The EEG data were without artefact and spikes^42^. EEG feature extraction was performed based on a sliding-window approach. The size of the window *(l)* was long enough to capture temporal patterns of the signal^56^, while considering the assumption of stationarity of the time series. The size of the window (*l*) is set pair to 5 or 10 s considering the different EEG features but is never below 60 s. We extracted 13 linear and non-linear features for each EEG signal using non-overlapping windows approach. We collected the data from all the 19 EEG electrodes for each patient during both wakefulness and sleep.

### Methodology in brain machine learning approach

The EEG features are used as input to the ANN classifier. We used a linear and non-linear methods to analyse data^25^ as reported in Supplementary Methods. An ANN approach was used for prediction of outcome after surgery using linear and non-linear features extracted from the pre-surgery EEG data as is shown in the Fig. 3. We trained 2 different topologies of ANN (A–B) and 9 different architectures (I–IX) of feedforward network with different numbers of hidden layers (HL) and different number of neurons (N) in each HL. The number of HL varied in the range of 1–3, while the number N in each HL varied based on the number of N in the first hidden layer (Tab. 2). We set a total of 54 confusion matrices, eighteen for each input SET. The maximum number of N was set following the empirical formula developed by Yotov et al.^57^. The number of neurons vary depending on the size of the training set. The output set consisted of two coded values, SF = 1 and NSF = 0. All networks were trained with a supervised approach using the Conjugate Gradient^58^. To verify the reproducibility of our results, all networks were trained 20 times using 70% of input patients randomly chosen as the training test, a random 15% of patients as the validation set and a random 15% of patients as testing set. A cross-validation scheme was used to train and test each classifier: the prediction accuracy was computed as the average of twenty iterations. To prevent overfitting and to improve performance and generalizability in the BML model we performed a feature selection: we divided our features in 3 different training sets, defined as a linear combination of different EEG features. We used the following features dataset:

1. SET 1: all features acquired by each channel;
2. SET 2: the average of all channel’s EEG features;
3. SET 3: EEG features statistically significant to the LR test after evaluating the correlation between the EEG pre-surgical features with the epileptic outcome (SF *vs* NSF).

**Table 2:** Different topology of ANN based on nine different artificial neural network architectures (from I to IX).

Architectures	Topology A	Topology B
I	n1	n1
II	n1 + n1	n1 + 1/2n1
III	n1 + n1 + n1	n1 + 1/2n1 + 1/3n1
IV	n2	n2
V	n2 + n2	n2 + 1/2n2
VI	n2 + n2 + n2	n2 + 1/2n2 + 1/3n2
VII	n3	n3
VIII	n3 + n3	n3 + 1/2n3
IX	n3 + n3 + n3	n3 + 1/2n3 + 1/3n3

They were defined by a specific combination of hidden layers (HL) and specific number of neurons (N).

Odds ratio (OR) in LR model was used to study the positive or negative correlation between the pre-surgical EEG features and the post-surgical outcome (SF *vs* NSF) to define the EEG features of SET 3; *p*-value below 0.05 were considered statistically significant. In implementing the LR model, the selected "response event" is always the SF condition. The set of features and the maximum number of neurons for each test is reported in Supplementary Table 3.

For each trained network, a confusion matrix was calculated based on the real output (seizure free or non-seizure free) and the one estimated on the randomly extracted testing set.

The mean 2 × 2 confusion matrix was then obtained by averaging the confusion matrixes of the trained ANNs for each iteration. A performance parameter (P) was calculated as the mean (%) of the elements on the diagonal of the mean confusion matrix, where 100% indicates the absence of misclassifications (Table 2).

**Figure 4:**
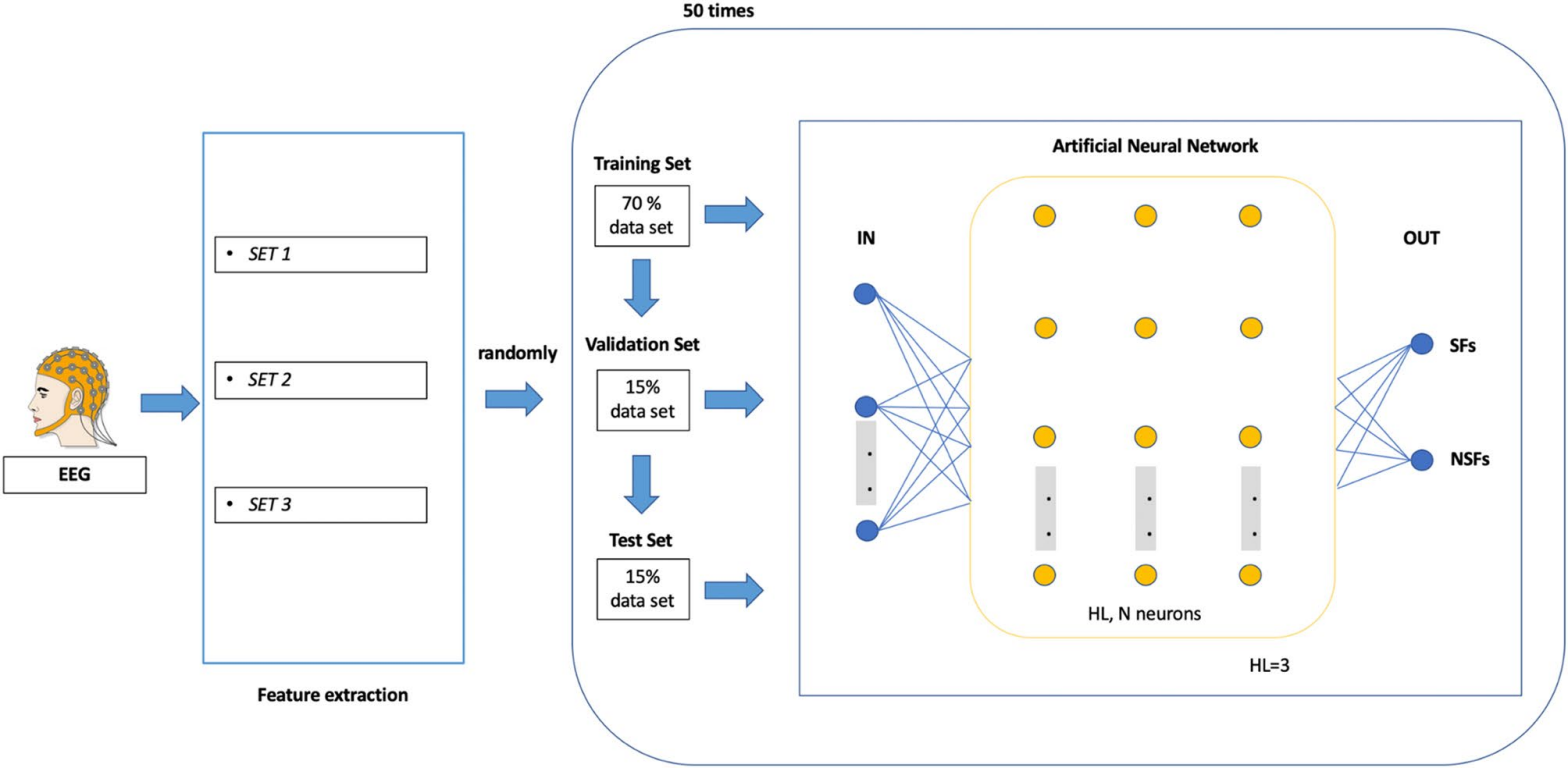
Study workflow and ANN architectures of the model. We used 3 Sets of EEG features to train and test the ANN models. We selected the 70% of data as training set and 30% for the validation and test sets. All architectures are composed of specific N and HL related to input data. Surgical outcome is dichotomous (SF–NSF). HD, hidden layer; N, neurons; SF, seizure freedom and drug freedom, NSF, not seizure freedom.

The mean square error (MSE) was calculated to select the most accurate training sets. The whole analysis process is illustrated in the Fig. 4.

The Wilcoxon Signed-Rank Test was used to rank the differences in performance between the best accurate classifier and the other ones for each architecture, and the *p*-values were found to be less than 0.05 with a confidence interval of 95%. We did not apply a *p*-value correction in the analyses stems from the exploratory nature of the study.

### Supplementary Information

Supplementary Information 1.

Supplementary Information 2.

## Data Availability

We declare that data supporting the results of this study are stored at Bambino Gesu’ Children’s Hospital internal repository and are available upon request to the corresponding author Dr. Nicola Specchio.
