# Author Correction: The value of linear and non-linear quantitative EEG analysis in paediatric epilepsy surgery: a machine learning approach

**DOI:** 10.1038/s41598-024-69706-8

**Published:** 2024-08-13

**Authors:** Mattia Mercier, Chiara Pepi, Giusy Carf-Pavia, Alessandro De Benedictis, Maria Camilla Rossi Espagnet, Greta Pirani, Federico Vigevano, Carlo Efsio Marras, Nicola Specchio, Luca De Palma

**Affiliations:** 1 Neurology, Epilepsy and Movement Disorders Unit, Bambino Gesù Children’s Hospital, IRCCS, Full Member of European Reference Network EpiCARE, Piazza S. Onofrio 4, 00165 Rome, Italy; 2 Department of Physiology, Behavioural Neuroscience PhD Program, Sapienza University, Rome, Italy; 3 Neurosurgery Unit, Bambino Gesù Children’s Hospital, IRCCS, Full Member of European Reference Network EpiCARE, 00165 Rome, Italy; 4 Neuroradiology Unit, Imaging Department, Bambino Gesù Children’s Hospital, 00165 Rome, Italy; 5 Department of Mechanical and Aerospace Engineering – DIMA, Sapienza University of Rome, Rome, Italy

Correction to: *Scientific Reports* 10.1038/s41598-024-60622-5, published online 13 May 2024

The Acknowledgements section in the original version of this Article was incomplete.

“This study was supported by #NEXTGENERATIONEU (NGEU) and funded by the Ministry of University and Research (MUR), National Recovery and Resilience Plan (NRRP), project MNESYS (PE0000006)—A Multiscale integrated approach to the study of the nervous system in health and disease (DN. 1553 11.10.2022). This study was also funded by “Precision medicine in epilepsy: definition of a predictive model of epilepsy outcome and creation of an automated tool based on machine learning.” Ricerca 5x1000 Bambino Gesu’ Children’ Hospital. Project code: 202205.”

now reads:

“This study was supported by #NEXTGENERATIONEU (NGEU) and funded by the Ministry of University and Research (MUR), National Recovery and Resilience Plan (NRRP), project MNESYS (PE0000006)—A Multiscale integrated approach to the study of the nervous system in health and disease (DN. 1553 11.10.2022). This study was also funded by “Precision medicine in epilepsy: definition of a predictive model of epilepsy outcome and creation of an automated tool based on machine learning.” Ricerca 5x1000 Bambino Gesu’ Children’ Hospital. Project code: 202205. This work was also supported by the Italian Ministry of Health with 'Current Research funds'.”

The original Article has been corrected.

