# Supplementary material for: The value of linear and non-linear quantitative EEG analysis in paediatric epilepsy surgery: a machine learning approach
Source: Sci Rep. 2024 May 13;14:10887. doi: 10.1038/s41598-024-60622-5 (PMC11091060; doi:10.1038/s41598-024-60622-5)
Supplement: Supplementary file 1 — Supplementary Information 1. [file 41598_2024_60622_MOESM1_ESM.docx]

**Supplementary methods**

We summarized the number, type and computational method of the extraction of linear and non-linear EEG features:

1. Linear

- Power Spectral Density (PSD): indicates the changes in the 5 frequency bands (alfa, beta, gamma, delta and total) of EEG signal measurable by applying Fourier transform. It was estimated as the mean of the PSD values ​​calculated over the 𝑁 5-seconds non-overlapping windows for each acquiring contact. The 6 relative powers (%) was calculated for each band compared to the total power spectrum.
- Hjorth (Mobility, Activity and Complexity): describe the characteristics of the EEG signal in terms of its amplitude (activity), slope (mobility) and slope spread (complexity)^1^. They were calculated over the N 10-seconds non-overlapping windows of EEG signal for each channel. Activity measures the variance of the time-varying data. Mobility calculates the ratio between standard deviations of the slope and of the amplitude of a signal per time unit. Complexity measures the similarity of the shape of a signal a pure sine waveform. The value of Complexity becomes close to 1 as the shape of signal gets more similar to a sine function waveform^2^

1. Non-Linear
   - Approximate Entropy (ApEn): is the logarithmic likelihood that the trends of the data patterns that are close to each other will remain close in the next comparison with next pattern. Is a measure of the data regularity^3^. The formula given by Pincus et al. has been used to extract ApEn from the 𝑁 10-seconds non-overlapping windows for each acquiring contact through the “ApEn” function of “entropyhub” matlab tool. The tolerance parameter *r* was set 0.2*times the standard deviation in order to avoid a significant contribution from noise in the EEG segments^3^,^4^. The embedding dimension 𝑚 and the time delay 𝜏 are set to 2 and 1 respectively.
   - Permutation Entropy (PermEn): is a complexity measure for time series based on comparing neighboring values of the series data. It describes the average rate of information associated with a stochastic source of data. It was estimated as the mean value of 𝑁 10-seconds non-overlapping windows through the “PermEn” matlab function developed by Matthew W.Flood^5^. It was computed with embedding dimension se to 3 and time delay set to 1
   - Lyapunov Exponent (LLE): is a measure of the dependence of the system on initial conditions. It can be defined as the average exponential rate of divergence or convergence. A negative exponent indicates that orbits approach a common fixed point, while a zero exponent means that orbits maintain their relative positions. If a positive LLE is achieved, it indicates the existence of chaos in that system^6^ . The LLE was estimated as the mean value of 𝑁 40-seconds non-overlapping windows through the “lyapunoveExponent” matlab function.
   - Hurst Exponent (H): is used to evaluate long range dependence and its dependence in a time series^7^. 0<H<0.5 means that with high probability, increasing value at time (*t*) follows by decreasing value at a time (*t+1*), similarly decreasing the value in time (*t*) follows by increasing value in time (*t+1*), this kind of waves also called anti-persistent; 0.5<H<1 means that increases in value will tend to increase and also decreases in value will tend to be followed by subsequent decreases (persistent); H=0.5, time series behaves as Gaussian noise or Brownian motion with a normal distribution with no long memory. was estimated with the Rescaled Range method and was obtained as the mean of the Hurst values ​​calculated over the 𝑁 4-seconds non-overlapping windows for each acquiring channel. It was used the “hurt_exponent” function for Matlab.

**References**

[1] Subha DP, Joseph PK, Acharya U R, Lim CM. EEG signal analysis: a survey. J Med Syst 2010;34:195–212.

[2] Oh S-H, Lee Y-R, Kim H-N. A Novel EEG Feature Extraction Method Using Hjorth Parameter. Int J Electron Electr Eng 2014;2:106–10.

[3] Acharya UR, Fujita H, Sudarshan VK, Bhat S, Koh JEW. Application of entropies for automated diagnosis of epilepsy using EEG signals: A review. Knowledge-Based Syst 2015;88:85–96.

[4] Pincus SM. Approximate entropy as a measure of system complexity. Proc Natl Acad Sci U S A 1991;88:2297–301.

[5] Flood MW. THE ENTROPYHUB GUIDE A user manual for the EntropyHub toolkit 2021;2.

[6] Iasemidis LD, Chris Sackellares J, Zaveri HP, Williams WJ. Phase space topography and the Lyapunov exponent of electrocorticograms in partial seizures. Brain Topogr 1990;2:187–201.

[7] Kannathal N, Acharya UR, Lim CM, Sadasivan PK. Characterization of EEG - A comparative study. Comput Methods Programs Biomed 2005;80:17–23.
