# Supplementary material for: The value of linear and non-linear quantitative EEG analysis in paediatric epilepsy surgery: a machine learning approach
Source: Sci Rep. 2024 May 13;14:10887. doi: 10.1038/s41598-024-60622-5 (PMC11091060; doi:10.1038/s41598-024-60622-5)
Supplement: Supplementary file 2 — Supplementary Information 2. [file 41598_2024_60622_MOESM2_ESM.docx]

**Supplementary Tables**

**Supplementary Table 1**: The results of descriptive statistic represent the mean values (SF and NSF) and standard deviation (SD) of the extracted linear and non-linear EEG features for both wakefulness and sleep condition between SF and NSF groups.

|  | ***state*** | ***NSF*** | ***SF*** | ***SD NSF*** | ***SD SF*** |
| --- | --- | --- | --- | --- | --- |
| **LLE** | *awake* | 0,19 | 0,19 | 0,19 | 0,01 |
|  | *sleep* | 0,19 | 0,19 | 0,19 | 0,01 |
| **ApEN** | *awake* | 0,65 | 0,63 | 0,63 | 0,11 |
|  | *sleep* | 0,79 | 0,81 | 0,81 | 0,12 |
| **PermEn** | *awake* | 0,65 | 0,66 | 0,66 | 0,03 |
|  | *sleep* | 0,69 | 0,70 | 0,70 | 0,04 |
| **Hurst** | *awake* | 0,54 | 0,68 | 0,68 | 0,02 |
|  | *sleep* | 0,72 | 0,89 | 0,89 | 0,02 |
| **Activity** | *awake* | 6,80 | 6,10 | 6,10 | 10,00 |
|  | *sleep* | 15,30 | 10,10 | 10,10 | 1,50 |
| **Mobility** | *awake* | 2,70 | 2,80 | 0,28 | 0,03 |
|  | *sleep* | 2,50 | 7,50 | 0,75 | 0,30 |
| **Complexity** | *awake* | 16,60 | 16,60 | 16,60 | 0,27 |
|  | *sleep* | 18,00 | 19,00 | 19,00 | 0,37 |
| **Psd_alfa** | *awake* | 0,39 | 0,18 | 0,18 | 0,02 |
|  | *sleep* | 0,81 | 0,22 | 0,22 | 0,03 |
| **Psd_beta** | *awake* | 0,19 | 0,13 | 0,13 | 0,01 |
|  | *sleep* | 0,55 | 0,90 | 0,90 | 0,10 |
| **Psd_gamma** | *awake* | 0,40 | 0,20 | 0,20 | 0,07 |
|  | *sleep* | 0,90 | 0,60 | 0,60 | 0,10 |
| **Psd_delta** | *awake* | 2,80 | 1,90 | 1,90 | 1,60 |
|  | *sleep* | 4,10 | 2,60 | 2,60 | 0,41 |
| **Psd_teta** | *awake* | 9,90 | 9,70 | 4,70 | 6,80 |
|  | *sleep* | 1,75 | 0,79 | 0,79 | 0,46 |

**Supplementary Table 2**: Descriptive characteristics of Histopathology findings on brain specimen.

| **Histology** | **Number of cases** |
| --- | --- |
| FCD I | 21 (17.1%) |
| FCD II | 22 (17.9%) |
| Hamartoma | 15 (12.2%) |
| Hs | 8 (6.5%) |
| Leat | 40 (32.5%) |
| Normal | 1 (0.8%) |
| Other | 13 (10.6%) |
| Rasmussen | 3 (2.4%) |

**Supplementary Table 3**: Number of sets of EEG features used in the predictive analysis and maximum number of neurons (Nmax) of the different ANN models. The complexity of the architectures is correlated to number of features used as input of ANN.

| ***SET*** | ***Features/patient*** | ***Nmax*** |
| --- | --- | --- |
| 1 | all features for each acquiring channel, wakefulness, and sleep EEG | 75 |
| 2 | mean of each feature on total channels, wakefulness, and sleep EEG | 36 |
| 3 | Mean of the statically significant features (p<0.05), wakefulness and sleep EEG | 9 |
